# Design of a novel multi-epitopes vaccine against *Escherichia fergusonii*: a pan-proteome based *in- silico* approach

**DOI:** 10.3389/fimmu.2023.1332378

**Published:** 2023-12-08

**Authors:** Taghreed N. Almanaa

**Affiliations:** Department of Botany and Microbiology, College of Science, King Saud University, Riyadh, Saudi Arabia

**Keywords:** *Escherichia fergusonii*, multi-epitopes vaccine, immunoinformatics, molecular dynamics simulation, reverse vaccinology

## Abstract

*Escherichia fergusonii* a gram-negative rod-shaped bacterium in the *Enterobacteriaceae* family, infect humans, causing serious illnesses such as urinary tract infection, cystitis, biliary tract infection, pneumonia, meningitis, hemolytic uremic syndrome, and death. Initially treatable with penicillin, antibiotic misuse led to evolving resistance, including resistance to colistin, a last-resort drug. With no licensed vaccine, the study aimed to design a multi-epitope vaccine against *E. fergusonii*. The study started with the retrieval of the complete proteome of all known strains and proceeded to filter the surface exposed virulent proteins. Seventeen virulent proteins (4 extracellular, 4 outer membranes, 9 periplasmic) with desirable physicochemical properties were identified from the complete proteome of known strains. Further, these proteins were processed for B-cell and T-cell epitope mapping. Obtained epitopes were evaluated for antigenicity, allergenicity, solubility, MHC-binding, and toxicity and the filtered epitopes were fused by specific linkers and an adjuvant into a vaccine construct. Structure of the vaccine candidate was predicted and refined resulting in 78.1% amino acids in allowed regions and VERIFY3D score of 81%. Vaccine construct was docked with TLR-4, MHC-I, and MHC-II, showing binding energies of -1040.8 kcal/mol, -871.4 kcal/mol, and -1154.6 kcal/mol and maximum interactions. Further, molecular dynamic simulation of the docked complexes was carried out resulting in a significant stable nature of the docked complexes (high B-factor and deformability values, lower Eigen and high variance values) in terms of intermolecular binding conformation and interactions. The vaccine was also reported to stimulate a variety of immunological pathways after administration. In short, the designed vaccine revealed promising predictions about its immune protective potential against *E. fergusonii* infections however experimental validation is needed to validate the results.

## 1 Introduction

*Escherichia fergusonii*, a relatively uncommon opportunistic pathogen in both humans and animals, is a rod-shaped, gram-negative bacterium that belongs to the genus *Enterobacteriaceae* (1). It is a peritrichous, non-spore-forming, and flagellated bacterium with a diameter between 0.8 and 1.5 mm and lengths between 2 and 5 mm (2). It was first isolated from samples of human blood in 1985 (3). The bacteria of the same genus demonstrated the strongest genetic resemblance to *E. coli*, with DNA hybridization revealing a 64% similarity (3). *E. fergusonii* is commonly found worldwide, and is suspected that this bacterium can enter the human body through food and water contaminated with infected feces (2). Furthermore, in a study, various strains of this bacterium were isolated from patients; including 2 strains from blood, 5 strains from urine, 1 strain from abdominal infection, 16 strains from feces, and 1 strain from other sites (4). Some strains of *E. fergusonii* have been associated with serious conditions in humans such as bloodstream infections, urinary tract infections, wound infections, and biliary tract infections (4).

In the context of antibiotic resistance (AR), *E. fergusonii* displayed resistance to a wide range of commonly used antibiotics. According to a recent study, ampicillin had proven to be particularly effective against the strains that caused these illnesses, while many showed resistance to gentamicin and chloramphenicol (5). Colistin is considered one of the most potent antibiotics for bacterial infections, and serves as the final line of defense in many cases (6). Even *E. fergusonii* had demonstrated resistance to colistin (7). A plasmid-borne resistance gene known as *mcr-1* was identified as the primary cause of widespread colistin resistance in many strains of *E. fergusonii* (8). In 2019, in Zhejiang, China, a complete sequence plasmid of *E. fergusonii* containing both *mcr-1* and ESBLs was discovered in chicken feces, suggesting that *E. fergusonii* may play a crucial role in transmitting *mcr-1* (9).

The effectiveness of antibiotics is at risk due to the rapid emergence of AR by bacterial pathogens worldwide (10). Inappropriate antibiotic use and the pharmaceutical industry’s lack of interest in discovering new medications have both been linked to AR (11). Efforts are required to devise new strategies to curb the spread of AR pathogens and effectively manage diseases caused by AR bacteria. To address this issue and combat bacterial infections promptly, vaccine production is one of the most effective approaches. History has shown that vaccines have successfully protected humanity from many deadly infections, such as chickenpox, hepatitis A, arboviruses, and tetanus (12). However, there is currently a challenge in the swift production of vaccines. Traditional vaccine development takes approximately 5-8 years to become available to the public. To overcome this issue and accelerate vaccine production, bioinformatics is playing a crucial role where it proposes a method known as *in-silico* vaccine designing (13). *In-silico* vaccine designing is a uniquely innovative approach in which the proteomes of microorganisms are utilized to map epitopes that are complementary to human immune receptors. When introduced into the body, these epitopes can evoke an immune response, directly leading to immune stimulation against the corresponding microorganism (14). This is swift and authentic approach where vaccine candidates can be designed in less time, minimizing the rate of AR pathogens and havoc in the community (15). Keeping the swiftness and accuracy of this approach in consideration, it is crucial for scientific community to use this methodology and design vaccine candidates against deadly bugs. Considering the increasing AR rate of *E. fergusonii*, there is a gap to use bioinformatics pipelines and design a MEV construct against it that could result in decrease in morbidity rate. Following the mentioned approach, a multi-epitope vaccine (MEV) construct against *E. fergusonii* was designed in this study by using a variety of bioinformatics, reverse vaccinology, immunoinformatics, and biophysics techniques.

The study was initiated by retrieving the complete proteome of 56 known strains of *E. fergusonii* from the NCBI database. These proteomes were collectively subjected to bacterial pan-genome analysis (BPGA) to identify the core proteome among the 56 strains. Furthermore, the core proteomes were processed to determine their subcellular localization, focusing on proteins that are more exposed to immune receptors and other immune cells, such as extracellular, outer membrane, and periplasmic proteins. The selected proteins underwent various analyses, including homology checks, assessment of transmembrane helices, and VFDB analysis to identify the most suitable proteins for epitopes prediction. The filtered proteins were then used to predict B-cell and T-cell epitopes. These epitopes were analyzed using various bioinformatics tools, and the selected epitopes were used in MEV candidate designing. Furthermore, the structure of the MEV candidate was predicted, and its binding efficiency to major human immune receptors, such as MHC-I, MHC-II, and TLR4 was evaluated. The docked complexes were simulated to assess their intermolecular binding stability versus time. Finally, the vaccine was expressed through *in-silico* cloning using the SnapGene tool. This methodology, as demonstrated in many other studies, has consistently yielded promising results, with vaccine candidates showing high efficiency in experimental phases (16, 17).

## 2 Research methodology

**Figure 1:**
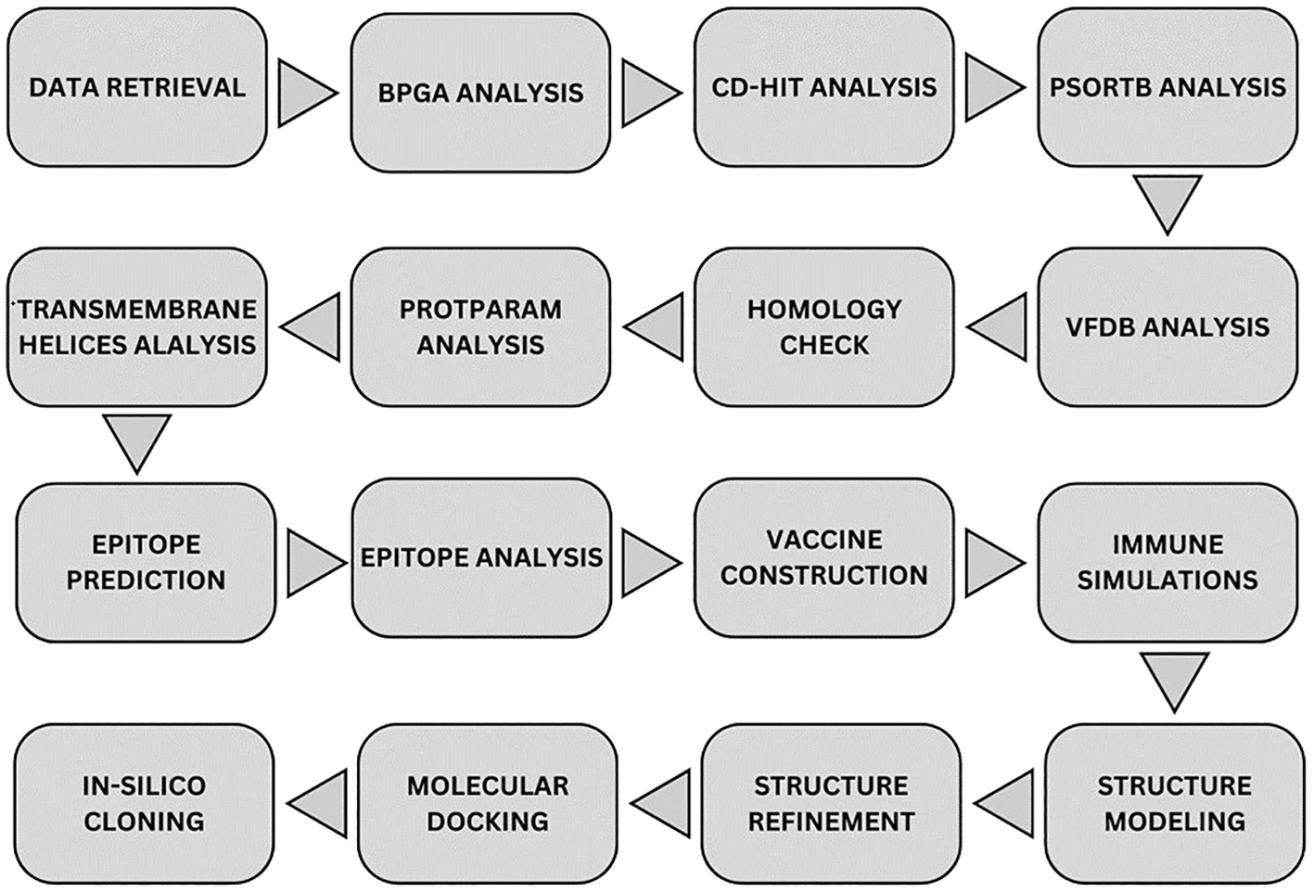
Overall methodology flow. Starting from top-left node, data of strains was downloaded from NCBI where it was processed to obtain core proteins via BPGA analysis. The non-redundant proteins were purified from the data via CD-HIT tool and the resulted data was processed for subcellular localization via PsortB tool. Obtained proteins were processed through four filtration tools in order to get the most virulent, non-homologous, most stable proteins. Epitopes were predicted and all the epitopes were analyzed through multiple filtration tools. MEV candidate was constructed and checked for immune simulation and the final structure was predicted for it. Structure was refined and was docked with the immune receptors in order to map its binding and simulations was carried out for each docked complex. Finally, the MEV candidate was re-expressed via *in-silico* cloning.

A step-by-step methodology was applied in the study to achieve the objectives as given in **Figure 1**.

### 2.1 *E. fergusonii* proteome retrieval

The study began with the retrieval of proteomic data for all strains of *E. fergusonii* (https://www.ncbi.nlm.nih.gov/datasets/genome/?taxon=564). This bacterium comprises 56 known strains that have been completely sequenced and are available in the NCBI database (18). All the proteomic data was downloaded and processed for BPGA analysis.

### 2.2 BPGA analysis

BPGA is a Perl-based pipeline used to organize the proteomes of different strains of bacteria into categories of core proteins (common proteins among strains), unique proteins, and accessory proteins (19). Our main objective was to identify the proteins that are shared across all strains of *E. fergusonii.*

### 2.3 CD-hit analysis

Redundant sequences are duplicates in the proteome and arise during the evolutionary process. As such, these sequences are not necessary for computational vaccine design strategies and therefore should be removed (20). The core proteome of all strains of *E. fergusonii* was submitted to the CD-HIT server, with a sequence identity cut-off value of 50% (21). CD-HIT is a fast and widely used tool for comparing and clustering peptide sequences, designed to remove all sequences that exhibit similarity greater than the cut-off value.

### 2.4 Subcellular localization

The filtered proteins underwent subcellular localization analysis. The entire set of proteins obtained from the CD-HIT tool was processed using the PsortB tool (22). This analysis was conducted to filter out the essential outer membrane, extracellular, and periplasmic proteins, as these protein types are most exposed to the immune system and can be the most effective vaccine candidates to induce immune responses (23). Proteins located elsewhere (cytoplasmic) were excluded from the study.

### 2.5 Virulence factor database analysis

The obtained proteins were further subjected to virulence factor analysis via the VFDB server (24). Each protein was individually assessed to determine if it qualifies as a virulence factor. The threshold criteria for a protein to be categorized as a virulence factor is a sequence identity of >30% and bit scores greater than 100 (25). This analysis was conducted because the virulence factors of a microorganism are essential for the pathogenesis (23). Therefore, selecting sequences that match the criteria is crucial in designing an effective vaccine (26). Proteins meeting the threshold criteria were chosen for further analyses, while the others were excluded.

### 2.6 Homology check

The filtered protein sequences underwent further processing for homology check. Each protein sequence was aligned with the proteomes of *Homo sapiens* and major human flora bacteria, namely *Lactobacillus johnsonni*, *Lactobacillus rhamnosus*, and *Lactobacillus casei*, using NCBI BLASTp tool (27). Proteins showing similarity ≥ 30% and a bit score of ≥ 100 to the mentioned organisms were excluded from the study. The remaining proteins were subjected to further analyses. This step was conducted to ensure that the selected protein sequences do not trigger autoimmune responses (28).

### 2.7 ProtParam analysis

The physicochemical properties of the filtered proteins were assessed using the ProtParam tool (29). Analyzing the physicochemical properties of proteins is crucial, as it aids experimentalists in their *in-vitro* and *in-vivo* examination of the designed vaccine construct (30). This analysis provides information on the molecular weight, number of amino acids, instability index, and aliphatic index of the obtained proteins. Additionally, it provides insight into the hydrophilic and hydrophobic properties of the proteins. Proteins were selected for further analyses based on their instability index. Proteins with an instability index greater than 40 were excluded from the study, as unstable proteins are not suitable for vaccine design (31).

### 2.8 Transmembrane helices analysis

Next, transmembrane helix analysis was conducted for the filtered proteins. This prediction indicates the likely cellular locations and is achieved through an algorithm known as N-best (or 1-best in this case). The algorithm aggregates all paths across the model that have the same placement and helical orientation. Only proteins containing 0 or 1 transmembrane helix were selected, as they are more amenable to protein purification in experimental analysis. This analysis was carried out using the online tool TMHMM 2.0 (32).

### 2.9 B-cell and T-cell epitope prediction

The immune prediction database (IEDB) was used to predict the possible B-cell epitopes in the selected proteins of *E. fergusonii* (33). To assess the effectiveness of these epitopes in binding to MHC-I and MHC-II, the obtained epitopes were further analyzed for T-cell epitopes (34). The peptides were selected based on low percentile scores because the lower the percentile score, the stronger the binding (35).

### 2.10 Epitopes analysis

The obtained epitopes underwent a series of analyses to filter and use them as part of the vaccine model. Antigenicity and toxicity were assessed for each epitope using Vexigen 2.0 (36) and the ToxinPred tool (37). Epitopes with an antigenicity value greater than or equal to 0.4 and no toxicity were further analyzed for solubility. Solubility for each epitope was determined using the Innovagen Peptide Calculator tool, and epitopes with good water solubility were processed further (38). The filtered epitopes were evaluated using the Allertop 2.0 tool to check for allergic responses, and only those epitopes that showed no allergenicity were considered for further analysis (39). Finally, the filtered epitopes were analyzed with the MHC-Pred tool to assess their binding efficiency to MHC receptors (40).

### 2.11 Epitopes designing and processing for vaccine candidate

The filtered epitopes were assembled to form a vaccine candidate in such a way that first, an adjuvant was attached via an EAAAK linker to the first epitope, followed by GPGPG linkers, each connecting two consecutive epitopes (41). Cholera toxin-B was added as an adjuvant to ensure that the vaccine candidate could induce a robust immune response both in good magnitude and intensity (42) The linkers were added to maintain the structural integrity of the vaccine and prevent self-complementary binding of the sequences (41). The physicochemical properties of the final construct were also predicted to evaluate its stability, theoretical isoelectric point (PI), half-life, and water solubility.

### 2.12 Immune simulation

The vaccine construct underwent immune simulation studies to assess the response of host immune system to the vaccine candidate. To accomplish this, the c-IMMSIMM tool was utilized (43). The server employs a position-specific score matrix (PSSM) and various other machine-learning techniques to predict and investigate epitope and immunological interactions (43).

### 2.13 Structure modeling and refinement

The iTESSOR (Iterative Threading ASSEmbly Refinement modeling tool) was employed to predict the three-dimensional structure of the final vaccine construct (44). iTESSOR employs a hierarchical method combining template-based fragment assembly, structural refinement, and threading (45). To predict a structure, it breaks the query sequence into fragments and utilizes all the structured sequences present in the Protein Data Bank (PDB) as templates against it (46). Upon prediction, it further refines the structure using specific algorithms. Because of this combined approach, iTESSOR is now at the forefront of protein structure prediction, allowing for precise structure prediction for a wide range of protein folds and sequences (47). The obtained structure was further refined using the Galaxy Refine tool to enhance stability (48). The stability of the vaccine structure was examined using Verify3D and PDBsum tools (49, 50).

### 2.14 Molecular docking and simulations

To assess the vaccine candidate’s ability to bind to the immune cell receptors, molecular docking was performed. The vaccine construct was docked with MHC-I (PDB ID: 1I1Y), MHC-II (PDB ID: 1KG0), and TLR4 (PDB ID: 4G8A) receptors using the ClusPro server (51). The complexes were then visualized using the UCSF Chimera software (52), and the types and number of interactions between the docked complexes were analyzed using the PDBsum tool (50). Understanding the atomic-level molecular behavior and characteristics of the docked complexes is possible to predict through molecular dynamics simulation (MDS). MDS provides data that complement experimental data regarding the dynamics, including structure, interaction energies, and atomic mobility (53). An online tool called iMOD was used to perform MDS, where dynamic parameters such as deformability factor, B-factor, Eigen-values, and variance of the docked complexes were calculated (54).

### 2.15 *In-silico* cloning

Finally, to confirm the cloning effectiveness of the designed vaccine candidate, it was re-expressed via *in-silico* cloning using SnapGene tool. Using *E. coli* as the expression system, the MEV’s codons were optimized according to the codon usage of the expression system. For this purpose, the Jcat tool was employed (55). The optimized and improved sequence was cloned into a specific vector known as pet28+(a) (56). This vector is mostly used in cloning approach because it consists of T7 promoter, His-tag fusion (polyhistadine which facilitates easy purification of the expressed protein), multiple-cloning-site and a selectable marker (57). The resulting data from this step will provide precise data for experimentalists to enable industrial-level production.

## 3 Results

### 3.1 Complete proteome retrieval of *E. fergusonii*

In this research work, a total 56 of completely sequenced proteomes of *E. fergusonii* were obtained from NCBI.

### 3.2 BPGA and CH-Hit analyses

**Figure 2:**
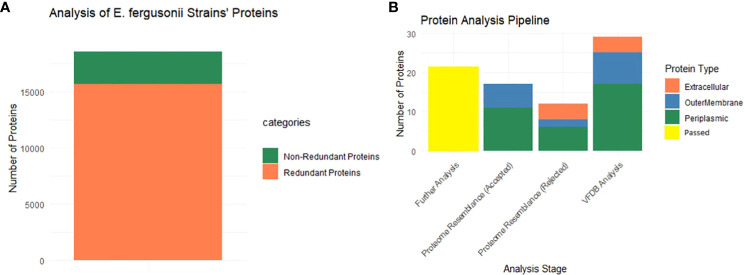
Graphical presentation. **(A)** This part shows that among the entire core proteins (entire multi-colored bar) 2911 proteins are non-redundant (dark green), and 15710 are redundant proteins. **(B)** Starting from the extreme right bar, the multi-colored bar shows the localized proteins that passed VFDB analysis (virulence proteins). Second bar shows the localized proteins that were passed in the homology check analysis (rejected word means that no similarity was shown with the proteome of the mentioned organisms) while the next plot is completely opposite of it. The last bar (yellow) shows the filtered proteins that are allowed for further analysis.

As mentioned, BPGA analysis filtered out the core proteome of all 56 strains of *E. fergusonii*. A total of 18,621 core proteins were filtered, which were further processed for CD-Hit analysis, resulting in the identification of 2,911 non-redundant proteins, while the remaining redundant proteins were excluded (**Figure 2A**). Redundant sequences are duplicates that arise due to duplication events during the evolutionary process. Therefore, for the computational vaccine design process, these sequences are not required. All the non-redundant proteins were subjected to subcellular localization and virulence analysis.

### 3.3 Subcellular localization, virulence and homology check

All 2,911 proteins were subjected to subcellular localization analysis resulting in the identification of 4 extracellular proteins, 8 outer membrane proteins, and 17 periplasmic proteins, while the remaining proteins were found to be cytoplasmic and were filtered for further analyses. The entire set of 29 proteins was then subjected to VFDB analysis, and all the proteins were included in the virulence factors list. Additionally, these proteins were aligned with the proteome of *H. sapiens* and major human flora bacteria, namely *L. johnsonni*, *L. rhamnosus*, and *L. casei*. None of the extracellular proteins showed any resemblance to the proteome of the mentioned organisms, while 2 outer membrane proteins and 6 periplasmic proteins displayed some resemblance to the proteome of the mentioned organisms and were thus rejected for further analyses. The graphical representation of these results is shown in **Figure 2B**. The remaining 21 proteins were processed further for the assessment of physiochemical properties

### 3.4 Predicting physicochemical properties and transmembrane helices

The physicochemical properties of the filtered proteins were checked using the ProtParam tool. The proteins with an instability index greater than 40 were classified as unstable and were removed from the list. Four proteins (two outer membrane proteins and two periplasmic proteins) were excluded, while the remaining proteins were stable. The remaining 17 proteins were processed for transmembrane helices analysis, and all of the proteins were within the threshold value (0-1). The final set of proteins is shown in **Supplementary Table 1**, and their properties are shown in **Table 1**. Furthermore, identity and functional enrichment analyses were carried out for all of the filtered proteins to determine their exact functions. The functional enrichment analysis of each protein revealed its molecular functions, roles in biological processes, and cellular components. All of the related information is shown in **Table 2**.

**Table 1:** Filtered proteins along with their properties.

PROTEINS/(Locality)	No. of Amino Acids	Molecular Weight	Instability Index	Aliphatic Index	TMHMM	VFDB	Homology Check
core/5905/1/Org1_Gene1275 (Extracellular)	163	16853.25	14.95	62.27	1	Virulence Factors	No Match
core/5991/1/Org1_Gene2681 (Extracellular)	160	17363.42	29.11	75.12	1		
core/6249/1/Org1_Gene2212 (Extracellular)	149	16529.45	23.07	77.25	0		
core/1874/1/Org1_Gene3952 (Extracellular)	879	97162.26	35.82	70.03	0		
core/234/1/Org1_Gene4318 (Outer membrane)	879	97162.26	35.82	70.03	0		
core/2441/3/Org3_Gene2670 (Outer membrane)	362	39207.87	21.36	75.22	0		
core/234/6/Org6_Gene3498 (Outer membrane)	877	96719.55	35.81	72.94	0		
core/234/44/Org44_Gene3892 (Outer membrane)	876	97282.15	36.19	76.03	0		
core/2286/1/Org1_Gene2488 (Periplasmic)	376	39740.17	33.01	94.6	0		
core/1236/1/Org1_Gene1255 (Periplasmic)	485	50780.95	33.09	80.9	1		
core/3965/1/Org1_Gene3693 (Periplasmic)	255	27745.65	28.85	78.78	1		
core/4549/1/Org1_Gene2707 (Periplasmic)	230	25121.82	37.25	100.48	0		
core/3965/3/Org3_Gene854 (Periplasmic)	257	28171.05	31.76	76.69	1		
core/4011/3/Org3_Gene3755 (Periplasmic)	260	29053.65	38.46	83.31	1		
core/725/4/Org4_Gene2970 (Periplasmic)	592	63511.01	26.71	78.97	0		
core/2888/11/Org11_Gene3787 (Periplasmic)	331	36003.95	35.21	98.34	1		
core/3965/22/Org22_Gene3561 (Periplasmic)	263	29315.3	35.64	80.8	1		

**Table 2:** Accession IDs along with protein length and functional annotations of filtered proteins.

Proteins Information			GO Annotation		
S/#	NCBI accession#	Protein Length	Biological Process	Molecular Function	Cellular Component
1	WP_000765273.1	152	amyloid fibril formation, cell adhesion, regulation of amyloid fibril formation, single-species biofilm formation	identical protein binding	pilus
2	WP_024256466.1	149	Cell adhesion, amyloid fibril formation, curli assembly, regulation of amyloid fibril formation, single-species biofilm formation	amyloidogenic domain that directs CsgA polymerization,	Cell outer membrane, pilus
3	WP_104920238.1	138	curli assembly, protein secretion by the type VIII secretion system	Assembly and substrate recognition of curli biogenesis system	cell outer membrane, curli secretion complex
4	WP_182211891.1	402	bacterial-type flagellum-dependent swarming motility	Unknown	bacterial-type flagellum basal body, bacterial-type flagellum hook, cytosol
5	WP_223665991.1	868	cell adhesion, pilus assembly	fimbrial usher porin activity	cell outer membrane
6	WP_024256413.1	351	monoatomic ion transmembrane transport	porin activity	cell outer membrane, pore complex
7	WP_148048017.1	866	pilus assembly	fimbrial usher porin activity	cell outer membrane
8	WP_181588385.1	865	pilus assembly	fimbrial usher porin activity	cell outer membrane
9	WP_002431495.1	365	bacterial-type flagellum-dependent cell motility, DNA damage response	structural molecule activity	bacterial-type flagellum basal body, distal rod, P ring, outer membrane-bounded periplasmic space
10	WP_000753932.1	474	chaperone-mediated protein folding, protein folding, protein quality control for misfolded or incompletely synthesized proteins, proteolysis, response to heat, response to oxidative stress, response to temperature stimulus	identical protein binding, peptidase activity, serine-type endopeptidase activity, serine-type peptidase activity	outer membrane-bounded periplasmic space, periplasmic space, plasma membrane
11	WP_182210933.1	244	cell wall organization, chaperone-mediated protein folding	unknown	outer membrane-bounded periplasmic space
12	WP_240828776.1	219	bacterial-type flagellum assembly	unknown	periplasmic space
13	WP_000465914.1	246	cell wall organization, chaperone-mediated protein folding	unknown	outer membrane-bounded periplasmic space
14	WP_223666952.1	249	post-transcriptional regulation of gene expression	RNA strand annealing activity, RNA strand-exchange activity	unknown
15	WP_105283498.1	581	glutathione biosynthetic process, glutathione catabolic process	glutathione hydrolase activity, hypoglycin A gamma-glutamyl transpeptidase activity, leukotriene C4 gamma-glutamyl transferase activity	unknown
16	WP_125400643.1	320	unknown	unknown	periplasmic
17	WP_181198759.1	252	cell wall organization, chaperone-mediated protein folding	unknown	outer membrane-bounded periplasmic space

### 3.5 Immune epitopes prediction

**Figure 3:**
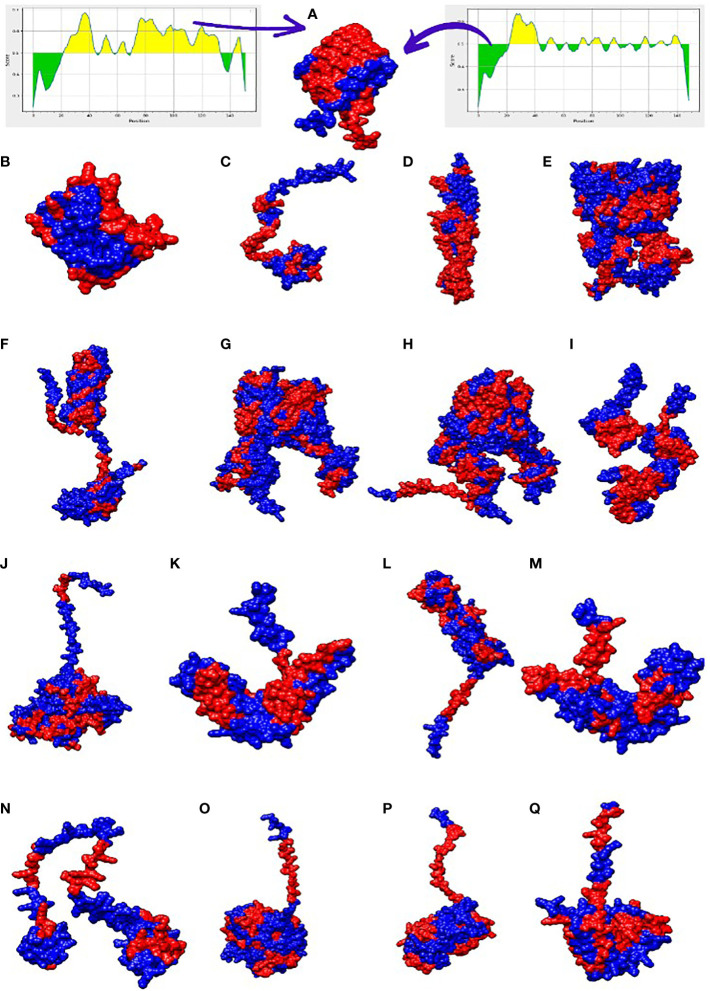
3D representation of the 17 filtered proteins. The graphs shown at top shows range of epitopes (residues) selected in the proteins (yellow region shows residues involved in B-cell epitopes while the green shows the unselected residues). The red regions in the structure represent B-cell epitopes while the blue portion represent the uninvolved residues. Three dimensional structures represent proteins in alphabetical order along with its NCBI accession IDs i-e WP_000765273.1 **(A)**, WP_024256466.1 **(B)**, WP_104920238.1 **(C)**, WP_182211891.1 **(D)**, WP_223665991.1 **(E)**, WP_024256413.1 **(F)**, WP_148048017.1 **(G)**, WP_181588385.1 **(H)**, WP_002431495.1 **(I)**, WP_000753932.1 **(J)**, WP_182210933.1 **(K)**, WP_240828776.1 **(L)**, WP_000465914.1 **(M)**, WP_223666952.1 **(N)**, WP_105283498.1 **(O)**, WP_125400643.1 **(P)**, WP_181198759.1 **(Q)**.

The selected proteins were prioritized for immune epitope prediction sequentially. This was achieved by B-cell epitope prediction followed by T-cell epitope prediction. B-cell epitopes were processed further to identify T-cell epitopes. Helper T lymphocytes stimulate B cells, macrophages, and cytotoxic T lymphocytes. Cytotoxic T lymphocytes, on the other hand, can directly recognize antigens (58). Conversely, B cells can develop into plasma cells, which produce antibodies (59). The lowest percentile score was used to select MHC-I and MHC-II epitopes in T cells, because the lower the score, the greater the binding potential. B-cell epitopes were predicted for each protein, and are shown in **Figure 3**. The number of amino acids and their sequence location in the proteins are listed in **Table 3**. The epitopes were processed further for T-cell epitope prediction (MHC-II and MHC-I). Numerous epitopes were predicted and processed for different filtration analyses to obtain the most appropriate epitopes for the final vaccine construct design.

**Table 3:** Detail of B-cell epitopes.

Protein	Protein length (size)	Epitope location (amino acid positions)	Epitope length (number of amino acids)
Prot1	152aa	23-45; 49-57; 61-67; 71-134; 144-149	23aa; 9aa; 7aa; 64aa; 6aa
Prot2	149aa	20-44; 53-55; 77-78; 85-88; 98-99; 120-121; 130-134; 139-144	25aa; 2aa; 4aa; 2aa; 2aa; 5aa; 6aa
Prot3	138aa	25-37; 46-70; 89-95; 116-122; 130-135	13aa; 25aa; 7aa; 7aa; 6aa
Prot4	402aa	29-52; 57-71; 97-225; 232-293; 305-309; 322-330; 333-354; 362-367; 392-397	24aa; 15aa; 129aa; 62aa; 5aa; 9aa; 22aa; 6aa; 6aa
Prot5	868aa	5-10; 31-65; 89-93; 108-133; 135-138; 161-177; 191-200; 211-234; 245-246; 257-266; 276-288; 315-317; 320-331; 343-348; 352-364; 375-382; 392-395; 421-426; 434-447; 451-462; 470-476; 486-508; 517-523; 534-544; 553-556; 567-573; 586-600; 605-614; 622-629; 636-647; 654-660; 666-676; 696-699; 718-725; 736-737; 748-756; 834-839; 848-856	6aa; 35aa; 5aa; 26aa; 4aa; 17aa; 10aa; 24aa; 2aa; 10aa; 13aa; 3aa; 12aa; 6aa; 13aa; 8aa; 4aa; 6aa; 14aa; 12aa; 7aa; 23aa; 7aa; 11aa; 4aa; 7aa; 15aa; 10aa; 8aa; 12aa; 7aa; 11aa; 4aa; 8aa; 2aa; 9aa; 6aa; 9aa
Prot6	351aa	24-26; 37-57; 76-93; 107-109; 126-145; 156-220; 228-238; 269-281; 305-329; 342-347	3aa; 21aa; 18aa; 3aa; 20aa; 65aa; 11aa; 13aa; 25aa; 6aa
Prot7	866aa	5-9; 32-63; 89-91; 104-132; 155-171; 187-194; 203-210; 213-229; 239-240; 253-260; 272-282; 303-304; 306-307; 309-311; 316-325; 340-347-349; 316-325; 337-340; 347-359; 369-377; 414-419; 431-438; 446-455; 468-470; 479-503; 511-518; 529-539; 546-552; 562-568; 581-594; 601-609; 617-624; 648-655; 663-672; 690-695; 711-721; 731-732; 743-751; 827-832	5aa; 32aa; 3aa; 29aa; 17aa; 8aa; 8aa; 17aa; 2aa; 8aa; 11aa; 2aa; 2aa; 3aa; 10aa; 4aa; 13aa; 10aa; 4aa; 13aa; 9aa; 6aa; 8aa; 10aa; 3aa; 25aa; 8aa; 11aa; 7aa; 7aa; 14aa; 9aa; 8aa; 12aa; 8aa; 10aa; 6aa; 11aa; 2aa; 9aa; 6aa; 7aa; 14aa; 9aa; 8aa; 12aa; 8aa; 10aa; 6aa; 11aa; 2aa; 9aa; 6aa; 11aa
Prot8	865aa	5-10; 32-48; 51-61; 86-88; 102-135; 141-145; 157-175; 189-196; 207-211; 218-230; 255-262; 273-284; 308-313; 318-327; 339-343; 348-361; 371-378; 348-361; 371-378; 387-389; 417-421; 431-442; 448-457; 469-473; 481-503; 515-520; 531-541; 549-554; 563-570; 583-610; 617-625; 633-644; 650-656; 664-672; 690-696; 716-723; 723-734; 745-752; 820-822; 845-855	6aa; 17aa; 11aa; 3aa; 34aa; 5aa; 19aa; 8aa; 5aa; 13aa; 8aa; 12aa; 6aa; 10aa; 5aa; 14aa; 8aa; 3aa; 14aa; 8aa; 3aa; 5aa; 12aa; 10aa; 5aa; 23aa; 6aa; 11aa; 6aa; 8aa; 28aa; 9aa; 12aa; 7aa; 9aa; 7aa; 8aa; 3aa; 8aa; 3aa; 11aa
Prot9	365aa	17-33; 47-57; 71-74; 76-79; 95-101; 113-116; 145-163; 173-182; 194-196; 206-216; 228-231; 242-249; 287-327	17aa; 11aa; 4aa; 4aa; 7aa; 4aa; 19aa; 10aa; 3aa; 11aa; 4aa; 8aa; 41aa
Prot10	474aa	20-40; 60-112; 181-184; 198-200; 213-221; 257-261; 283-287; 232-330; 358-360; 382-408; 420-427	21aa; 53aa; 4aa; 3aa; 9aa; 5aa; 5aa; 8aa; 3aa; 27aa; 8aa
Prot11	244aa	61-71; 85-86; 100-104; 118-129; 147-156; 194-207; 214-224; 234-240	11aa; 2aa; 5aa; 12aa; 10aa; 14aa; 11aa; 7aa
Prot12	219aa	17-25; 47-71; 107-110; 117-163; 186-192	9aa; 25aa; 4aa; 47aa; 7aa
Prot13	246aa	5-6; 64-73; 87-89; 101-106; 120-129; 150-159; 193-205; 215-226; 237-242	2aa; 10aa; 3aa; 6aa; 10aa; 10aa; 13aa; 12aa; 6aa
Prot14	249aa	65-70; 84-86; 98-105; 118-124; 143-153; 185-194; 204-212; 224-226; 234-246	6aa; 3aa; 8aa; 7aa; 11aa; 10aa; 9aa; 3aa; 4aa
Prot15	581aa	27-46; 124-143; 161-167; 183-184; 186-187; 193-123; 241-244; 261-263; 312-321; 343-363; 368-390; 335-358; 529-533; 545-547; 573-577	20aa; 20aa; 7aa; 2aa; 2aa; 31aa; 4aa; 3aa; 10aa; 21aa; 23aa; 24aa; 5aa; 3aa; 5aa
Prot16	320aa	24-33; 74-109; 126-136; 148-154; 164-175; 224-249; 252-256; 269-273; 300-301	10aa; 36aa; 11aa; 7aa; 12aa; 26aa; 5aa; 5aa; 2aa
Prot17	252aa	5-11; 74-84; 98-99; 112-118; 132-141; 160-169; 201-211; 222-232; 243-248	7aa; 11aa; 2aa; 7aa; 10aa; 10aa; 11aa; 11aa; 6aa

Epitope location with start and end residue along with its length is shown respectively.

### 3.6 Epitopes prioritization phase

The obtained epitopes were finally processed for multiple analyses to obtain the most acceptable epitopes for our vaccine construct. Antigenicity, allergenicity, solubility, MHCpred, and toxicity analyses were performed, and the filtered epitopes are shown in **Table 4**. The non-antigenic epitopes were eliminated from the selected epitopes, as the vaccine should contain epitopes that have the potential to induce antibody production. Epitopes that are less soluble in water, exhibit a toxic character and produce allergic reactions, were further eliminated.

**Table 4:** Filtered epitopes along with their sequential analysis.

EPITOPES	ANTIGENICITY	ALLERGENICITY	SOLOBILITY	TOXICITY	MHCpred
AVDQTASNS	Antigen	Non-Allergen	Soluble	Non-Toxic	≤ 100 nm
SATLDQWNS					
YNDDFGIET					
DGTTTNTGR					
VKTFDASNA					
TKDNTWQVY					
GARDIDVNR					
DIDVNRYSK					
ADNAILQKR					
RENAAQDCL					
KLADNAILQ					
DDKTYSGQS					
NVQKNSDSL					
TSSDHSRQY					
STSSDHSRQ					
NGPTHENQL					
PAPAPAPEV					
FNFNKATLK					
AYNQGLSER					
DYWAGNNNR					
NLDKDSEDV					
DKDSEDVAS					
PDIHSENAV					
EQKLEPQSM					
PEQKLEPQS					
QKLEPQSMR					
NGIEGAEMS					
QSSQNQVDS					
PPKMSDADA					
GAPRSVSGA					
AISLRDIAP					
DSKKSLTSH					
KVPWQALTN					
IDINKAKPS					
IRPGKLAPY					
ERKLQRLYI					
SENADPSTL					

### 3.7 Vaccine candidate design

**Figure 4:**
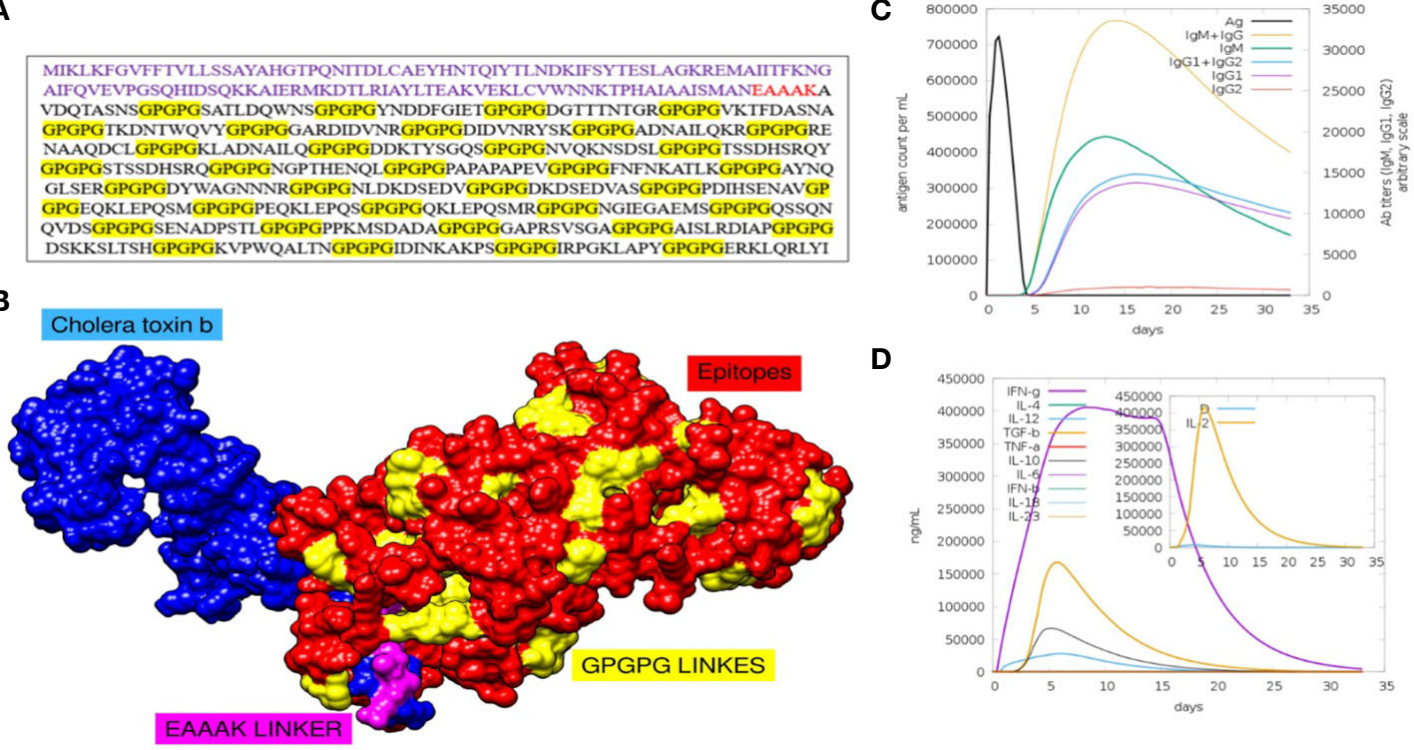
**(A)** Sequence of final MEV construct. **(B)** Predicted structure of MEV construct along with its adjuvants and different linkers. **(C)** Number of different antibodies produced against the vaccine in specific number of days are plotted. **(D)** Number and types of cytokines with respect to number of days is plotted.

Following the completion of the above investigation, 37 distinct epitopes were selected from the list of combined epitopes before filtration. One of the key issues was solved by combining various types of specified epitopes using specific GPGPG linkers to form a multi-epitope-based vaccine construct. Additionally, the cholera toxin B subunit adjuvant and the epitopes peptide were linked using the EAAAK linker. GPGPG linkers were inserted between epitopes because they can prevent junctional folding and effectively trigger an immune response involving T-helper cells (60). EAAAK is a stiff, stable α-helical peptide linker with an intramolecular hydrogen bond and a closed-packed backbone. Thus, in a fusion protein, the EAAAK linker serves as a domain spacer (61). Furthermore, linkers help epitopes to unite and produce a considerable structure having polytope conformation (62). Because cholera toxin B significantly increases the synthesis of mucosal IgA and other immunological responses, it was used as an adjuvant (63). This is because it is non-toxic and can bind to the monosialotetrahexosylganglioside (GM1) receptor, which is present on cell membranes and in the cytosols of a range of cells, including B cells, macrophages, dendritic cells, gut epithelial cells, and antigen-presenting cells (64). Similarly, the adjuvant used is safe and generates potent immune responses that are specific to the antigen it is associated with (65). The MEV construct and its important properties are shown in **Figure 4A** and **Table 5**, respectively.

**Table 5:** Important properties of vaccine along with its half-life in human body.

	Molecular Weight	No. of amino acids	Instability Index	Theoretical PI	GRAVY	Antigenicity	Half Life	Aliphatic index
**Vaccine construct**	63930.06	637	28.82	5.51	-0.825	Antigen	>20h	49.09

### 3.8 Immune simulation analysis and structure modelling

To assess whether the epitopes would be capable of generating sufficient immunity, the immunological responses to the multi-epitope vaccination were evaluated using the C-ImmSim server (66). This method can also be used to determine the development of immunological interactions between the epitopes and their specific targets. The ability of the MEV construct to elicit potent immune responses was evident via this approach. After 35 days of exposure of the human immune system to the highest dose of vaccine antigen, a C-immune simulation analysis revealed an increase in the generation of adaptive responses in the form of IgG and IgM-antibodies (**Figure 4C**). Similarly, it was noted that interferon-gamma and IL-2 production was greater than 400000 counts per ml for nearly 35 days (**Figure 4D**). At last, the MEV construct was modelled in **Figure 4A**. For this, the amino acid of the final MEV construct was uploaded to iTESSOR tool in FASTA format, and the structure was predicted using an ab initio modelling strategy (67). A total of five structures were predicted and the structure having the highest confidence score was selected.

### 3.9 Structure refinement and stability analysis

**Figure 5:**
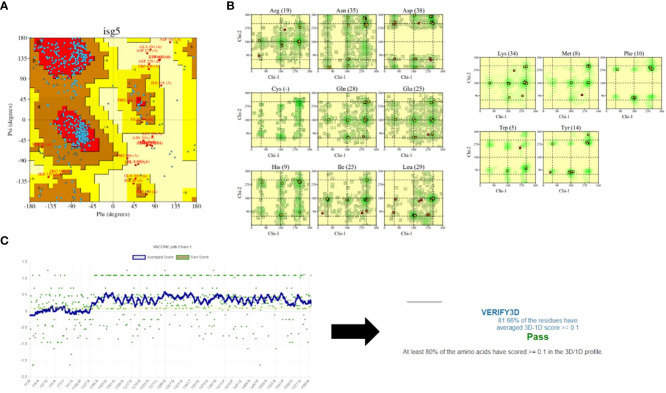
Stability analysis: **(A)** Ramachandran plot shows maximum of residues (blue dots) in the allowed regions while very few residues (red dots) in the disallowed regions. **(B)** shows the dihedral angles of each residues in MEV construct. **(C)** shows that maximum of residues of MEV construct lies among the averaged line that clarify that the structure is almost stable.

The predicted model was further refined using the GALAXY refinement tool to minimize, relax, and stabilize the structure by removing high-energy interactions (68, 69). The stability of the predicted model was examined using the PDBsum and Verify3D tools (49, 50). These tools predicted the stability using plots and graphs. The plot generated by the PDBsum tool is known as the Ramachandran plot, where the amino acids of the MEV construct are divided and placed in different regions of the plot, each of which represents a specific level of stability (70). This plot consists of four regions: most favoured regions (red), additional allowed regions (brown), generously allowed regions (yellow), and disallowed regions (pale). The residues of the MEV construct were placed in these regions and combined stability was calculated. The placement of residues in these regions is based on the Phi and Psi angles of each residue, which are plotted on X-axis and Y-axis of the plot. Upon analysis, most of the residues of the MEV construct were placed in the allowed region (78.1%), followed by the additional allowed regions (16.7%), the generously allowed region (1.7%), and the disallowed region (3.6%) **Table 6**. The occurrence of more residues in the allowed regions and fewer in the disallowed region indicated that our MEV construct is stable (**Figure 5A**). Furthermore, plots based on dihedral angles for each residue were shown separately, clearly indicating the occurrence of each residue across the plot. It shows that most of the residue’s dihedral angles are within the supported range, while only a few residues (indicated by yellow and red spots) have controversial angles, collectively clarifying the stability of the MEV construct (**Figure 5B**). Secondly, Verify3D also clarifies the stability of the MEV construct, showing that the majority of residues (81.66%) lie close to the average score line (**Figure 5C**).

**Table 6:** Statistics of ramachandran plot.

Parameter/Region	Vaccine	
	No. of Residues	Percentage
Most favored regions (A, B, C)	498	78.1%
Additional allowed regions (a, b, l, p)	106	16.7%
Generously allowed regions (~a, ~b, ~1, ~p)	11	1.7%
Disallowed regions (XX)	22	3.6%
Non-glycine and non-proline residues	420	100%
End-residues (excl. Gly and pro)	12	
Glycine residues	117	
Proline residues	88	

This table illustrates maximum number of residues in the most favoured regions of the plot that represents maximum stability of the vaccine.

### 3.10 Protein-protein molecular docking

**Figure 6:**
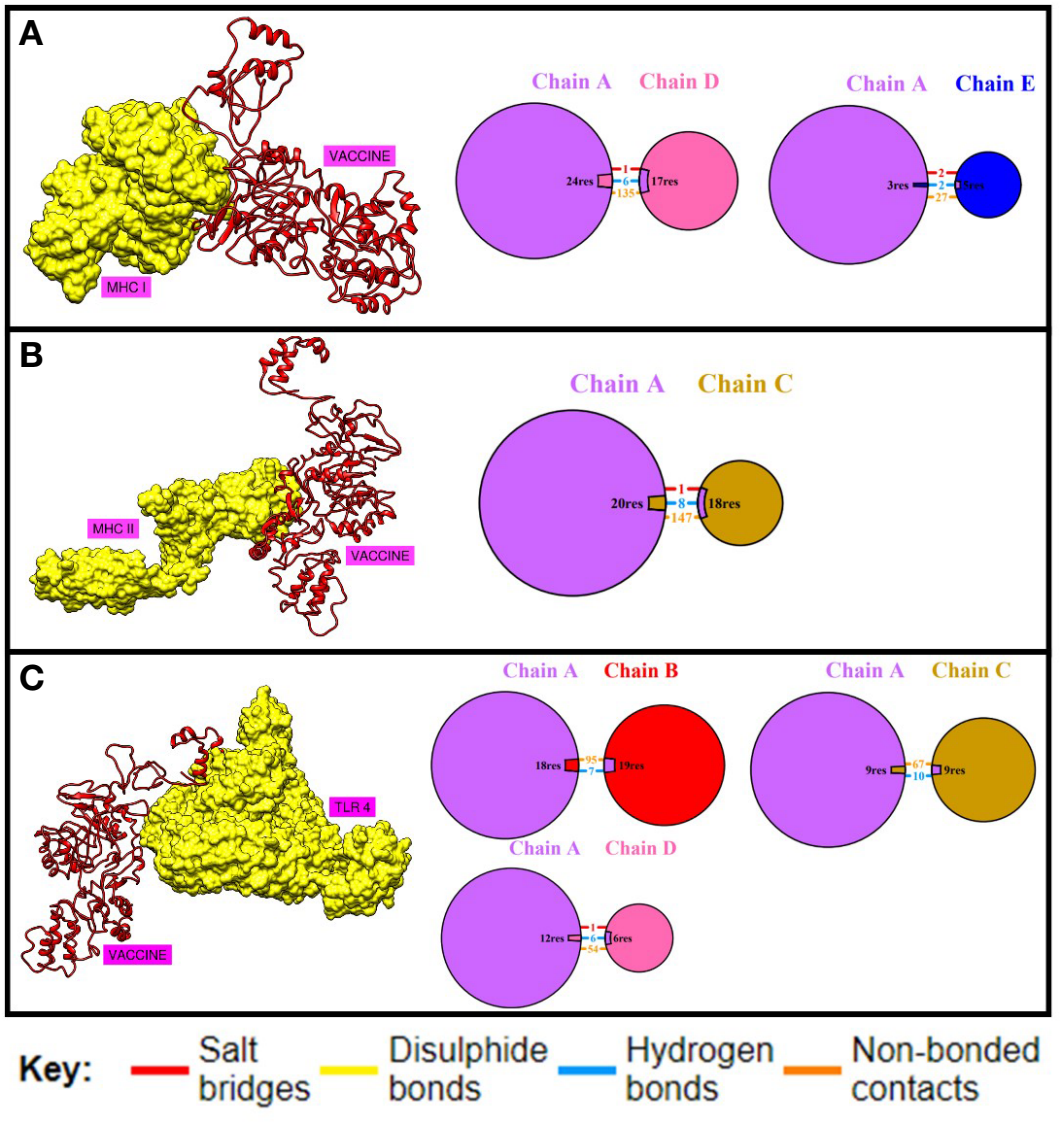
Binding analysis. **(A)** shows the number and type of interactions between the chains of MHC-I and MEV construct. **(B)** this shows the number and type of interactions between MHC-II and MEV construct while **(C)** shows the binding and interactions between TLR04 and MEV construct respectively.

Docking is an important strategy for evaluating the binding efficiency of two molecules. This approach was used to check the binding affinity of the MEV construct with the immune receptors i-e MHC-I, MHC-II, and TLR-4. Three-dimensional structures of immune receptors were retrieved from the Protein Data Bank, and each was separately docked with the MEV construct (51). Binding energies were then calculated (51). Furthermore, the types and number of interactions were also checked (50). The binding of the vaccine with TLR4, MHC-I, and MHC-II was efficient **Table 7** and the number of interactions was also maximum **Figure 6**. Further, hydrogen bonds were also present between the complexes, which further illustrate maximum bindings. The structure of docked complexes and the number and types of interactions between the complexes are shown in **Figure 6**.

**Table 7:** Top 1 clusters of the docked complexes along with their number of members (residues) involved in the interaction and its binding energies are shown.

DOCKED COMPLEXES	CLUSTER	MEMBERS	BINDING ENERGY
TLR 4–VACCINE	TOP 1	58	-1040.8 kcal/mol
MHC I–VACCINE	TOP 1	57	-871.4 kcal/mol
MHC II–VACCINE	TOP 1	80	-1154.6 kcal/mol

Lower the binding energies represents maximum bindings.

### 3.11 Molecular dynamic simulations of the docked complexes

**Figure 7:**
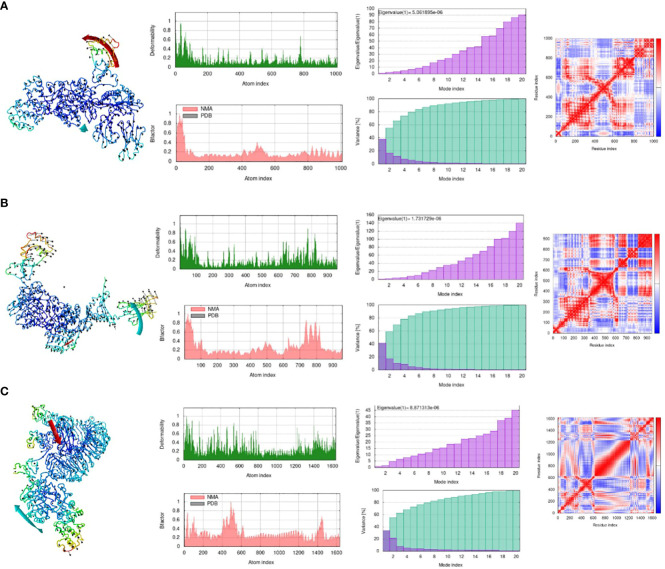
Molecular Dynamic Simulation. **(A)** MHC-I-MEV complex, **(B)** MHC-II-MEV complex, **(C)** TLR4-MEV complex. Deformability plot, eigenvalue, B-factor, variance plot, covariance matrix analysis and elastic network model. (Analysis in sequential manner).

Online simulation studies were performed for all the docked complexes separately using the iMOD tool to check the behavior of the docked complexes (54). Simulation results shown in **Figure 7** reveal that all the docked complexes showed considerable deformability values, which illustrates that most of the residues of the complex are deforming and showing movement from their actual position. This indicates that greater deformability results in active binding. Second, the eigenvalues of all the complexes show the lowest values, which indicates that less energy is required to deform the structures, which correlates with effective binding. Variance is the opposite of eigenvalues, with high values indicating low difficulty in deforming the structure. The B-factor of all the complexes represents high values, which also correlates with the deformability of complexes. Red, white, and blue colours are used in the covariance matrix to indicate the graph’s correlated, uncorrelated, and anti-correlated motions, respectively. In short, the molecular dynamics investigation clearly showed that our complexes had a fair deal of deformability and displayed an acceptable eigenvalue, indicating effective and uniform binding to its immune receptors.

### 3.12 *In-silico* cloning and codon optimization

**Figure 8:**
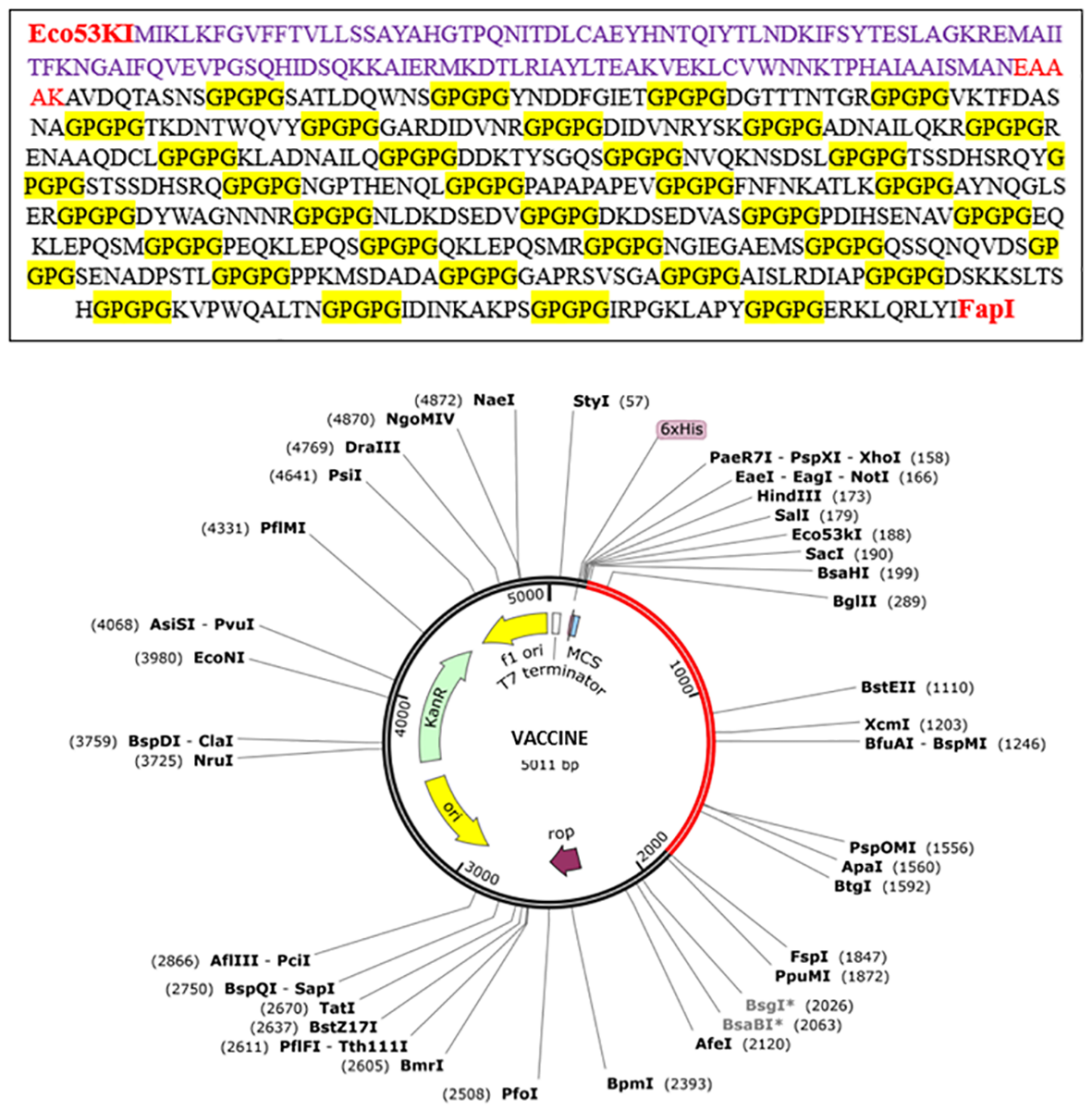
*In-silico* cloning of the vaccine construct. Red part shows the vaccine construct cloned into the vector pet28a(+) with restriction sites Eco53kI and FspI used. Translated sequence of the red area is also shown.

The predicted vaccine was finally subjected to *in-silico* cloning because it assured us that it was the finest vaccine candidate to evoke immune responses and prevent *E. fergusonii* infection in individuals. For cloning, the MEV construct was optimized using the codon adaptation tool (55) according to the expression system *E. coli K-12* (71). Codon optimization was performed to match the expression system used because the efficiency of codons varies among different organisms due to specific codon usage patterns. The MEV construct was successfully cloned into a special vector named pet28a(+) with modified restriction sites using the SnapGene tool, and the results are shown in **Figure 8** (72).

## 4 Discussion

AR among pathogenic microorganisms is increasing at a rapid pace (10). As a result, most of the known antibiotics available are becoming less effective, leading to an increase in morbidity and mortality rates (73). This situation is primarily a consequence of antibiotic misuse by the population (74). *E. fergusonii* is a minor pathogenic bacterium, that is responsible for causing UTIs, biliary infections, and hemolytic uremic syndrome (HUS). It was once easily treated with common antibiotics, but over time, this bacterium has developed AR properties. Recently, it had shown resistance to one of the most potent antibiotics, colistin, leading to a public health crisis (8). The main objective of this research was to design an MEV based on the core proteome of all strains of *E. fergusonii*. The goal is to confer immunity against this bacterium, preventing its pathogenesis within the host’s body (75). MEV constructs have been designed by many researchers against many pathogens considering their entire proteomes. For example, a recent study used superantigens of *Staphylococcus aureus* to construct a MEV construct (76). Another study focused on designing an MEV against *Helicobacter pylori* (77). Furthermore, MEV constructs have been designed against the spike proteins of coronaviruses, yielding promising results (78)

The field of vaccine design is seeing significant advancements due to reverse vaccinology, and the availability of proteomics data and development in artificial and machine learning techniques (79). Additionally, the successful application of bioinformatics technologies is advantageous in comparison to conventional vaccine design (80). The designing of the MEV construct has also shown promising experimental outcomes that include a study by (17) where the MEV construct against influenza-A showed robust production of neutralizing antibodies in mice models. Further, (16) proposed a study in which an MEV construct designed against *Leishmania infantum* proteins showed abundant production of interferons and interleukins in BALB mice when administered.

Following the methodology of the study, core proteomes from the known strains of completely sequenced *E. fergusonii* were obtained, and CD-Hit analysis resulted in the generation of non-redundant sequences from the core proteomes. Vaccines can only be effective if they contain portions of the bacteria that are more readily exposed to the body’s immune cells and are highly virulent (contributing to pathogenesis), resulting in rapid immune responses (81, 82). Following this concept, exposed proteins were filtered out from the non-redundant proteins and were further prioritized for a virulence check. Same concepts were used by different researchers like, (83) designed a MEV against *Mycobacterium ulcerans* strain Agy99 by considering its chromosome-encoded virulence proteins and (84) produced an epitope-based vaccine against *Mycobacterium* spp. by utilizing its extracellular 85B protein, respectively. (85) designed an effective MEV candidate based on major virulence factors of *Clostridium perfringens*, i.e., alpha-toxin, NetB, and metallopeptidase protein (NAM). Thereafter, the highly virulent and exposed proteins’ sequences were processed through homology checks by aligning the sequences with the proteomes of *Homo sapiens* and important human normal flora bacteria. The aligned sequences were removed from the study, and the remaining sequences were allowed to be processed further. (86) also used the same homology check protocol for *Chlamydia psittaci* proteins when designing MEV.

Physiochemical properties analysis of proteins is one of the important steps in MEV designing (87). Upon examining, 21 selected proteins exhibited high stability, with instability index values ranging from 14 to 36. Four proteins were excluded due to instability index values exceeding 40. The remaining proteins had molecular weights between 149 and 879 kDa and high aliphatic indices, indicating their ability to withstand high temperatures and suitability for vaccine design.

The active acquired immune responses are extremely specialized and focused on eliminating pathogens or preventing their development (88). Adaptive immunity produces memory B-cells that identify the organism after the initial recognition of subsequent encounters (89). Vaccination is based on such immunological recall of adaptive immunity (90). The primary function of the B and T lymphocyte cells in the adaptive immune system is to produce cellular immunity against invader organisms that are reliant on antibodies. Multiple B-cell epitopes were predicted for the proteins that were further processed for T-cell epitopes prediction in the form of MHC-I and MHC-II receptors. Because epitopes with the lowest percentile scores demonstrate the greatest affinity to receptors, they were given priority and chosen based on their low percentile scores (75).

Approximately 324 different epitopes were predicted, which were then processed for various analyses. The epitopes were filtered based on the following criteria: non-toxicity in living organisms, maximum solubility in water, maximum antigenicity, no allergic responses, and maximum binding with HLA-DRB1*0101 alleles. An essential immune system protein is made according to directions from the HLA-DRB1 gene. The human leukocyte antigen (HLA) complex is a cluster of genes that includes the HLA-DRB1 gene. The HLA complex helps the immune system distinguish between proteins produced by the host’s cells and those produced by foreign invaders, such as viruses and bacteria. The major histocompatibility complex (MHC), a gene family found in many species, is represented in humans by the HLA complex (91). The MHC class II subset of MHC genes includes the HLA-DRB1 gene (92). Certain immune system cells have proteins on their surface that are produced according to the instructions of MHC class II genes (93). These proteins bind to peptides, or short fragments of proteins, outside the cell. These peptides are presented to the immune system by MHC class II proteins. When the immune system detects peptides that are foreign to it, such as viral or bacterial peptides, it launches an attack against the invaders (94).

The final vaccine candidate was prepared by linking the epitopes together with the help of linkers that make the vaccine more stable and avoid self-binding between it. These linkers make the vaccine intact and expose the epitopes to bind the immune receptors sufficiently. The vaccine was also linked with an adjuvant known as cholera b toxin so that immune activation could be enhanced. The physicochemical properties of the final vaccine construct were checked which showed that it has 637 amino acids and has a molecular weight of 63.930 kDa which is far more in the acceptable range. The instability index showed a value of 28.82 which clearly illustrates that the vaccine is quite stable. The vaccine candidate was slightly acidic having a smaller number of positive and more negative residues and the theoretical PI was 5.51. It showed a hydrophilic nature due to a negative GRAVY value i-e -0.825 which is good for a normal protein to act efficiently inside the body (87). Antigenic analysis showed that the vaccine is highly antigenic and its half-life inside the body is greater than 20 hours which is normal for maximum proteins. The aliphatic index also had a maximum value of 49.09 which shows that the vaccine can withstand high temperatures.

The vaccine’s three-dimensional structure was obtained and refined for enhanced stability. Ramachandran plot and Verify3D analyses were performed to assess stability. The plot features four quadrants (I, II, III, and IV) where Phi angles (angles between alpha carbon and nitrogen) and Psi angles (angles between alpha carbon and carboxyl carbon) were plotted on the Y and X axes, respectively plus red and blue dots represent amino acid residues. The plot shown below **Figure 5A**) exhibits a common feature: it comprises four regions distinguished by color. The red region, known as the most favored region, represents amino acids with angle values (Phi and Psi) that lack steric hindrance, indicating that their molecules do not obstruct each other (95). In simpler terms, a greater abundance of amino acids in favored regions suggests enhanced flexibility for docking. The brown region, an additional permitted area, also signifies protein flexibility. The yellow region, or generously allowed region, poses high hindrance to Phi and Psi angles; an increased presence of amino acids here indicates processing challenges. The pale yellow, or disallowed region, strictly restricts Phi and Psi angle rotation due to steric hindrance (96). A higher proportion of amino acids in this region suggests that the protein cannot be processed. For the vaccine, the majority of amino acids were present in the red region, yielding a value of 78.1%, which is far more favorable and indicative of maximum stability.

Maximum binding of the vaccine with important receptors of the immune cells is important to evoke a robust immune response. The vaccine candidate was docked with the immune receptors i-e TLR4, MHC-I, and MHC-II respectively. The binding energy was quite high and a maximum number of amino acids were involved in the binding of both proteins. Examining the docked complex of MEV-TRL4, the binding energy was -1040.8 kcal/mol, the number of non-bonded interactions was 216, hydrogen bonds were 23, and the disulfide bond was 1. Number of residues involved in these interactions were 58. The docked complex of MEV-MHC I illustrates a binding energy of -871.4 kcal/mol and several non-bonded and bonded interactions and were i-e 162 non-bonded, 8 hydrogen bonds, and 3 disulfide bonds, and many residues involved in hydrophobic interactions were 57. The docking complex of Vaccine-MHC II resulted a binding energy of -1154.5 kcal/mol showed 147 non-bonded interactions, 8 hydrogen bonds, 1 disulfide bond, and 80 residues involved. Simulation studies for the complexes also show promising results which revealed acceptable deformability and B-factor values which means that residues of both proteins show deformability from their actual position at the time of interactions. Eigenvalues and variances show that less energy is required to displace the residues from their origins showing easy deformability of the complexes.

Being a promising vaccine candidate fulfilling all the requirements, *in-silico* cloning was assured to produce this vaccine and make it available to humanity. To achieve this, the principles of recombinant DNA technology were followed, involving finding a suitable vector to insert the desired vaccine sequence and using an expression system to produce it. For this, the pet28a(+) vector and *E. coli* were used as the expression system because it has the highest multiplication rate.

## 5 Conclusions

In this work, we have developed a multi-epitope vaccine construct against the pathogenic bacteria *E. fergusonii*, which causes a range of diseases in people, utilizing several bioinformatics techniques. This bacterium has acquired antibiotic resistance as a result of improper use of antibiotics and the absence of a recognized immunization. The sole means of preventing illnesses brought on by *E. fergusonii* was vaccination. For the components of the immune system, the MEV sequences utilized in the produced vaccine have the best adhesion efficacy and are antigenic, non-allergic, highly soluble, and non-toxic. To elicit a strong immune response against MEV, an adjuvant was added and these obtained epitopes were connected by linkers. The immunological responses to the planned vaccine were highly concerning, and the vaccine itself was fairly stable. The results and predictions of the current study should expedite the process of developing a vaccination against *E. fergusonii* and provide insights that may help in this regard. The study’s findings will also help save time and money, and experimental vaccine scientists will be helped by the *in-silico* vaccine design to produce a vaccine against *E. fergusonii* infections for both therapeutic and preventive uses.

## Data availability statement

The original contributions presented in the study are included in the article/**Supplementary Material**. Further inquiries can be directed to the corresponding author.

## Author contributions

TA: Conceptualization, Data curation, Formal analysis, Funding acquisition, Investigation, Methodology, Project administration, Resources, Software, Supervision, Validation, Visualization, Writing – original draft, Writing – review & editing.
