# Supplementary material for: Design of a novel multi-epitopes vaccine against Escherichia fergusonii: a pan-proteome based in- silico approach
Source: Front Immunol. 2023 Dec 8;14:1332378. doi: 10.3389/fimmu.2023.1332378 (PMC10739491; doi:10.3389/fimmu.2023.1332378)
Supplement: Supplementary file 1 [file Table_1.docx]

**Supplementary Files**

**Design of a Novel Multi-Epitopes Vaccine against *Escherichia fergusonii*: A Pan-proteome Based *In- Silico* Approach**

**Taghreed N. Almanaa^1^**

^1^Department of Botany and Microbiology, College of Science, King Saud University, Riyadh 11451, Saudi Arabia. [talmanaa@ksu.edu.sa](about:blank)

* **Correspondence:**

[talmanaa@ksu.edu.sa](about:blank)

**Supplementary Table 1.** Finalized proteins along with their amino acid sequence.

| **Proteins (Location)** | **Sequence** |
| --- | --- |
| core/5905/1/Org1_Gene1275 (Extracellular) | MKLFKVAAIAAIVFSGSVLAGTVPQFGGGGGHNPGNGNNNGPNSELNIYQYGGGNSAVALQTDAKNSDLTITQHGGGNGADVGQGSDDSSIDLLQRGFGNSATLDQWNSKDSIMKVKQYGGGNGAAVDQTASNSQVNVTQVGFGNNATAHQY |
| core/5991/1/Org1_Gene2681  (Extracellular) | MKNKLLFMMFTILGAPGIATATDMANSEYNFAVNELSKSSLNQAAIIGQYGTDHSAQIRQGGSKLLSVISQEGGSNRAKIDQSGDYNLAYIDQSGSANDASISQGSYGNTAIILQKGSGNKANITQYGTQKTAIVVQRQSNMAIRVTQR |
| core/6249/1/Org1_Gene2212  (Extracellular) | MRVQHAVVLLMLISPLSWAGNMTFQFRNPNFGGNPNNGAFLLNSAQAQNSYKDPSYNDDFGIETPSALDNFTQAIQSQILGGLLTNINTGKPGRLVTSDFIVDIANRDGQLQLNITDRKTGKTSTIEVSGLQTNSTDF |
| core/1874/1/Org1_Gene3952  (Extracellular) | MAFSQAVSGLNAAATNLDVIGNNIANSATYGFKSGTASFADMFAGSKVGLGVKVAGITQDFTDGTTTNTGRGLDIAISQNGFFRLVDSNGSVFYSRNGQFKLDENRNLVNMQGLQLTGYPAAGTPPTIQQGANPTNISIPNTLMAAKTTTTAAMQINLNSSDPLPTVKTFDASNADSYNKKGSVTVFDSQGNAHDMSVYFVKTKDNTWQVYTQDSSDAASQATQATTLEFNANGTLVNGAMANNIKTSAINGAVPATFSLSFLNSMQQNTGANNIVATTQNGYKPGDLVSYQINDDGTVVGNYSNEQTQLLGQIVLANFANNEGLASEGDNVWSATQASGVALLGTAGTGNFGTLTNGALEASNVDLSKELVNMIVAQRNYQSNAQTIKTQDQILNTLVNLR |
| core/234/1/Org1_Gene4318  (Outer membrane) | MKIPLDRKKNQRSPLACLIACSLLPCTQNVWGANETVEFDNTFLMGAGARDIDVNRYSKGNATLPGRYDVSVFINNQASANLKIEFIELDPKHAAQPCISTNTLLQLHIRQPEKLADNAILQKRENAAQDCLNVEVAIPQSSVAYNSNDQRLDISMPQIWLQRTYANYVDPSLWDEGINAAMLSYTLNGWRSESPNQTTETSYAGLMGGINLWGWHFRSHGNYSWDKEDGGSFEFQDKYLQRDIAALRSQLVMGETNTTGETFDSVPIRGLRLYSESRMLPPTLASYAPVIRGVANSNAKVTITQNGYKIYETTVPPGPFAIDDLTPAGFGADLEVTITESDGSKRTFSQAYSSVIQMMRPGVGKWDISAGEVNKAELQDKPNLLQGTYYYGLSNTFTGYTGLQMTDNGYWAGLLGVGMNTSLGAFSFDVTQSHAEIPDDKTYSGQSYRISWNKYFAPTETSLNIAAYRYSTENYLGLNDALTLINSANHPDEEGYSGIVSYARMKNQFSVSINQTLKSDERDYGSFYVNGTWTDYWVTNESQSSFAFGYSNAFNWASYSVSLQRTYDEDNKKDDSVYLSVTIPLDKLLGRDSHQGGFKTLNTSVNSDMKGSSQYNANASGYSQDNRWSYSVNTAYNVQKNSDSLKSISGYTSYESPWGTFSGSASTSSDHSRQYGLSTDGGFVLHRHGLTFSNDSFTDSDTLALINAPGAKGARINFGNSTIDRFGYGVTSSLSPYRENTVALDVNDLDNDVELKNTSSVAIPRQGSVIFSHFDTDTGRSAILNLLRSNNLPVPFASDITDEKGTSIGTVGQGSQAFVCGIANSGELTVTWYEKNSPQQCRIHYQIPSSPTMSGKTIVLNAVPCTQQ |
| core/2441/3/Org3_Gene2670  (Outer membrane) | MKKTAIAIAVALAGFATVAQAAPKDNTWYTGAKLGWSQYHDTGFIDNNGPTHENQLGAGAFGGYQVNPYVGFEMGYDWLGRMPYKGSVENGAYKAQGVQLTAKLGYPITDDLDIYTRLGGMVWRADTKAHNNVTGESEKNHDTGVSPVFAGGVEWAITPEIATRLEYQWTNNIGDANTIGTRPDNGLLSLGVSYRFGQGEAAPVVAPAPAPAPEVQTKHFTLKSDVLFNFNKATLKPEGQAALDQLYSQLSNLDPKDGSVVVLGYTDRIGSDAYNQGLSERRAQSVVDYLISKGIPADKISARGMGESNPVTGNTCDNVKQRAALIDCLAPDRRVEIEVKGIKDVVTQPQA |
| core/234/6/Org6_Gene3498  (Outer membrane) | MYTLTHQKSRLPKKTLLAVCCAFFYSANGAATESVEYDSSFLMGAGASTIDIKRYSQGNPTPPGVYNVRVFVNEQSVASLELPFVDIGENSAAACITRKNLAQLHIKQPESPITLIARKGEEGDCLDIKKSIEQAEVRYDGGEQYLEISVPQAYVYKTYGGYVDPSLWESGINAATLSYSLNAYHSDSYNDNRDSIYGAFNTGLNFGVWHLRARGNYNWSQDNGNSFDFQDRYLQRDIPAIRSQVIVGDAYTTGETFDSVNVRGMRLYSDSRMLPSVLASYAPTIRGVANSNAKVTVTQNGYKIYESTVPPGEFVIDDLSPSGFGSELVITIEEADGSKRSFTQPFSSVVQLQRPGVGRWDISAGQIIDDSLRHEPNMAQASFYYGFNNLFTGYTGVQITDNDYMSGLLGLGINTSIGAFAVDVTHARTEIPDDKTYQGQSYRITWNKLFEATSTSFNLAAYRYSTENYLGLHDALALIDDANHLSSDESKDTMRTYSRMKNQFTVSVNQPLNFAYEDYGSLFLSGSWTDYWAGNNNRTEYNVGYSKSVFWGSFSVNLQRSWNEDGDKDDAMYLNVSVPLENIFGGKRKSSGFRNLNTQFNTDFNGSHQLNVSSSGNSEDNLIGYSVNTGYNLDKDSEDVASVGGYLSYDSIWGGFSASASASTDNRQQYSVSTDGGFVLHSGGLTFTNNSFGSNDTLVVIKAPGAKGARVNNGTDEIDRWGYAVSSSLSPYRENRVGLNIETLENDVELKSTSATTVPRSGSIILASFETDQGRSAVLNISASNGKSIPFAAEVYQDEIMIGSMGQGGQAFVRGINDNGELIVRWFENSQAMNCKLHYQLPAQPETLGSTNTLLLNNLTCKLVNH |
| core/234/44/Org44_Gene3892  (Outer membrane) | MQKITITHPCNLTQIATLCSLLYSSCSISAEHVEYDHTFLMGRDASNIDLSRYSEGNPTLPGIYDVNVYVNDQPVINQSIPFVVIDGKKNAQACITQKNILQFHITQPDIHSENAVLLERDEELGDCLNLTEIIPQSSVRYNVNDQRLDIDVPQAWVMKNYQNYVDPSLWENGINAAMLSYNVNGYHSESPDRKNDSLYASFNGGVNLGAWRMRASGNYNWMNDSGSDYNFQNRYLQRDLASLRSQLIIGESYTTGETFDSVSIRGVRLYSDSRMLPPALASFAPVIHGVANTNAKVTITQSGYKIYETTVPPGAFTIDDLSPSGYGSDLIVTIEEADGSKRTFSQPFSSVVQMLRPGVGRWDISGGQVLKDDIQDEPNLFQASYYYGLNNYLTAYTGIQLTDNNYTSGLLGLGLNTSFGAFSFDVTHSNVRIPEDKTYQGQSYRLSWNKLFEDTSTSLNIAAYRYSTQNYLGLNDALTLIDEVKHPEQKLEPQSMRNYSRMKNQVTVSINQPLKFEKKDYGSFYLSGSWSDYWASGQNQSNYSIGYSNSASWGSYSISAQRSWNEDGETDDSIYLSFTIPIEKLLGTEHRDSGFQNIDTQLNSDFKGNNQLNISSSGYNDNNRISYSVNTGYTMNKSTDDLSYIGGYASYESPWGTLAGSVSASSDNSRQFSLNTDGGFVLHSGGLTFSNDSFSDSDTLAVVQAPGAKGARINYGNSTVDRWGYGVTSALSPYHENRIALDINDLENDVELKSTSTVAVPRQGSVIFAGFETVQGQSAIMNIKRSDGRNIPFAADIYDEQGNNIGNVGQGGQAFVRGISQQGNIQIKWLEAGKPVNCLAHYQQNAEAEKIAQTIIINGINCQIQ |
| core/2286/1/Org1_Gene2488  (Periplasmic) | MIKFLSTFMLLLVTTVVQAERIRDLTSVQGVRQNSLIGYGLVVGLDGTGDQTTQTPFTTQTLNNMLSQLGITVPAGTNMQLKNVAAVMVTASLPPFARQGQTIDVVVSSMGNAKSLRGGTLLMTPLKGVDSQVYALAQGNILVGGAGASAGGSSVQVNQLNGGRITNGAIIERELPSQFGAGNTLNLQLNDEDFAMAQQIADTINRARGYGSATALDSRTIQVNVPSGNSSQVRFLADIQNMQVNVTPQDAKVVINSRTGSVVMNREVTLDSCAVAQGNLSVTVNRQANVSQPDTPFGGGQTVVTPQTQIDLRQSGGSLQSVRSSANLNNVVRALNALGATPMDLMSILQSMQSAGCLRAKLEII |
| core/1236/1/Org1_Gene1255  (Periplasmic) | MKKTTLALSALALSLGLALSPLSATAAEATPAASAQQMPSLAPMLEKVMPSVVSINVEGSTTVNTPRMPRNFQQFFGDDSPFCQEGSPFQSSPFCQGGPGGNGGGQTQKFMSLGSGVIIDAEKGYVVTNNHVVDNANVIKVQLSDGRKFDAKMVGKDPRSDIALIQIQNPKNLTAIKMADSDALRVGDYTVAIGNPFGLGETVTSGIVSALGRSGLNVENYENFIQTDAAINRGNSGGALVNLNGELIGINTAILAPDGGNIGIGFAIPSNMVKNLTSQMVEFGQVKRGELGIMGTELNSELAKAMKVDAQRGAFVSQVMPNSSAAKAGIKAGDVITSLNGKPISSFAALRAQVGTMPVGSKLSLGLLRDGKPVTVNLELQQSSQNQVDSSSIFNGIEGAEMSNKGKDQGVVVNNVKTGTPAAQIGLKKGDVIIGANQQAVKNIAELRKILDSKPSVLALNIQRGDSTLYLLMQ |
| core/3965/1/Org1_Gene3693  (Periplasmic) | MCQFIVSTAFVASTAVFSLAAQADIVLSGTRIIYNADQKDVTLRMENKGARPLLVQTWIDTGSENADPSTLKVPFTVTPPVSRVDGKKGQTVKIAWNASRTLPADHESVYWFNALEVPPKMSDADAKGKNILQLAFRTRIKLFYRPTGLAGNPADAPKQLTWRLQSSAGKVVLQATNPTPYHVSFSSITLTSGGNKYTVETSMVEPKSSAEMVVKGAPRSVSGATVEYSAINDFGGGIDGKVKL |
| core/4549/1/Org1_Gene2707  (Periplasmic) | MRRFCPPVAAIVLLLSTYCHAQDLNSQLNNWFAARLAGLSDNVVVTLRTPPERQPKCDNPALSMSNNSRLWGNINVLAQCGNIRYYLQVNVQASGNYVVAAAPITRGGVVNASNVTLKRGRLDQLPPRTVLEVAQVQDAISLRDIAPGQPVQLNMLRQAWRIKAGQRVQVIAQGDGFNVNAEGQALNNAAVAQTARVRMLSGQVVSGIVGADGNILINL |
| core/3965/3/Org3_Gene854  (Periplasmic) | MFFNTKHTAALCFATCVAFSSSAIADIVISGTRVIYKSDQKSVSVRLENKGNNPLLVQSWLDTGDDNAEPGSITVPFTATPPVSRIDAKRGQTIKLMYTASSVLPKDRESVFWFNVLEVPPKPDAATTTNQSLLQLAFRTRIKLFYRPEGLKGNPSDAPLALKWTWATAGGQTALRVANPTPYYVSFSSGDLETSGKRYPLDVKMIAPFSNDVIKVNGMSGKSSSAKVHFYAINDFGGAIEGNATL |
| core/4011/3/Org3_Gene3755  (Periplasmic) | MNKMKSLLTATGMICLLGICNFAQATVSPDRTRIIFNASNKSATVRLTNQSKIDPYLAQSWIEDASGKKTRDYISTLPPMERIEANEQVQIRLMALASLNNLPQDRETVFYYNVREIPPRAKEQNVMQIAMQSRLKLFWRPKAIELKDGEAVPVQKVAVARSANGLTLNNPTPYHITVGYIGTNGKTLMPGTDSIMVTPFGTATQQIKNLPATFLLGFVGDYGGLEMFKIDCNSVQSLCQTSPAKKGKL |
| core/725/4/Org4_Gene2970  (Periplasmic) | MIKPTFLRRVAIATLLSGCCFSAVAAPPAPPPVSYGVEEDVFHPVRAKQGMVASVDATATQVGVDILKKGGNAVDAAVAVGYALAVTHPQAGNLGGGGFMLIRSKNGNTTAIDFREMAPAKATRDMFLDDQGNPDSKKSLTSHLASGTPGTVAGFSLALDKYGTMPLNKVVQPAFKLARDGFIVNDALADDLKTYGSEVLPNHENSKAIFWKEGEPLKKGDKLVQANLAKSLEMIAENGPDEFYKGTIAEQIAQEMQKNGGLITKEDLAAYKAVERTPISGDYRGYQVYSMPPPSSGGIHIVQILNILENFDMQKYGFGSADAMQIMAEAEKHAYADRSEYLGDPDFVKVPWQALTNKAYAKSIADQIDINKAKPSSEIRPGKLAPYESNQTTHYSVVDKDGNAVAVTYTLNTTFGTGIVAGESGILLNNQMDDFSAKPGVPNVYGLVGGDANAVGPNKRPLSSMSPTIVFKDGKTWLVTGSPGGSRIITTVLQMVVNSIDYGMNVAEATNAPRFHHQWLPDELRVEKGFSPDTLKLLEAKGQKVALKEAMGSTQSIMVGPDGELYGASDPRSVDDLTAGY |
| core/2888/11/Org11_Gene3787  (Periplasmic) | MRLAPLYRNALLLAGLLLSGISAVQAADWPRQITDSRGTHTLESPPQRIVSTSVTLTGSLLAIDAPVIASGATTPNNRVADDQGFLRQWSKVAKERKLQRLYIGEPSAEAVAAQMPDLILISATGGDSALALYDQLSTIAPTLIINYDDKSWQSLLTQLGEITGHEKQAAERIAQFDKQLTAAKEQIKLPPQPVTAIVYTAAAHSANLWTPESAQGQMLEQLGFTLAKLPAGLNASQSQGKRHDIIQLGGENLAAGLNGESLFLFAGDQKDADAIYANPLLAHLPAVQNKQVYALGTETFRLDYYSAMQVLERLKALFES |
| core/3965/22/Org22_Gene3561  (Periplasmic) | MSHNYTIARSAYVFLCKKNVSVLFISTLLTSAPAFADIIISGTRIIYFADKKDVNVRLENKGNRPLLIQNWLDTGDDNADPSQIKVPFTSTPPVSRIEPKRGQTVKVMFTGTTQLSADRESVFWFNVLEVPPKPKDAESDKNLLQLAFRTRIKLFYRPSGLQGEPAEAPAKLTWKLNNSQLQVQNPTPYYVSFNDVTLESGTRSYKVDSSMVAPFAQASFDVTGLPGSVSSGKVIYKAINDYGGNIDGSASL |
